# Indications of Clinical and Genetic Predictors for Aromatase Inhibitors Related Musculoskeletal Adverse Events in Chinese Han Women with Breast Cancer

**DOI:** 10.1371/journal.pone.0068798

**Published:** 2013-07-19

**Authors:** Jingxuan Wang, Kangping Lu, Ying Song, Li Xie, Shu Zhao, Yunxuan Wang, Wenzhou Sun, Lei Liu, Hong Zhao, Dabei Tang, Wenjie Ma, Bo Pan, Qijia Xuan, Hang Liu, Qingyuan Zhang

**Affiliations:** Department of Medical Oncology, The Third Affiliated Hospital of Harbin Medical University, Harbin, China; Sudbury Regional Hospital, Canada

## Abstract

**Background:**

Women with breast cancer treated with aromatase inhibitors (AIs) may experience musculoskeletal symptoms that lead to discontinuation of effective therapy. The purpose of the current study is to evaluate the clinical and genetic predictors for AIs-related musculoskeletal adverse events(MS-AEs).

**Methodology and Principal Findings:**

We recruited 436 postmenopausal Chinese Han women receiving adjuvant AIs therapy for early-stage hormone-sensitive breast cancer. Patients completed a self-administered questionnaire assessing the presence of musculoskeletal symptoms that started or worsened after initiating AIs. 27 single nucleotide polymorphisms (SNP) of ESR1, ESR2 and PGR were analyzed by Sequenom MassARRAY assays and /or PCR-based TaqMan assays.Of the 436 enrolled women, 206 cases experienced musculoskeletal symptoms.Patients who received taxane chemotherapy were more than two times more likely than other patients to have AIs-related MS-AEs. Genetic assay had showed that only two ESR1 SNPs, rs2234693 and rs9340799 were associated with AIs-related MS-AEs.TT genotype and the T allele in rs2234693 was statistically significantly lower in AIs-Related MS-AEs group than controls (P = 0.001; P = 9.49E-7). The frequency of AA genotype and the A allele in rs9340799 was higher (P = 2.20E-5; P = 3.09E-4).

**Conclusions and Significance:**

Our results suggested that prior taxane-based chemotherapy was the clinical predictor, while rs2234693 and rs9340799 were the genetic predictors for AIs-related MS-AEs.

## Introduction

The third-generation aromatase inhibitors (AIs), anastrozole, letrozole and exemestane, which prevent the conversion of androgens to estrogens by reversibly or irreversibly inhibiting the aromatase enzyme, have shown benefits over tamoxifen, in both adjuvant and metastatic treatment for postmenopausal hormone-responsive breast cancer women [1]–[5]. However, because AIs profoundly reduce the already low circulating oestrogen levels in postmenopausal women by a further 80–90% compared with tamoxifen, this agent is associated with a modest increase of the deleterious effects on musculoskeletal system, i.e. arthralgia, osteoporosis, and bone fractures [6], [7]. These MS-AEs are also an important reason of treatment discontinuation [8], [9], three national longitudinal databases found that adherence to adjuvant anastrozole therapy decreased from 69% to 78% in year 1 and 50% to 68% in year 3 [10], [11].

Three large randomised controlled trial (ATAC, BIG 1–98 and TEAM) demonstrated that development of MS-AEs in women receiving AIs adjuvant therapy might predict a reduced risk of breast cancer recurrence [12], while it was no association in MA.27, a large phase III trial comparing the anastrozole with exemestane as adjuvant therapy for early breast cancer [13]. We could not say that AIs-related MS-AEs is bad or good now, but interruption of treatment is not indeed a good thing.

The etiology of AIs-related MS-AEs is poorly understood, which challenges the development of effective management strategies. A variety of pharmacological interventions to prevent or treat AIs-related MS-AEs have been largely ineffective [6]–[7], [14]–[16]. AIs prevent peripheral estrogen production, a further 80–90% decrease compared with tamoxifen or natural menopause. Estrogen has positive effects on the regulation of bone turnover, acquisition of peak bone mass and inhibition of bone loss.Estrogen deficiency after menopause has been linked to an increase in several chronic inflammatory conditions, including osteoporosis and osteoarthritis(OA), so some researches believed that AIs-related MS-AEs were associated to excessive estrogen deprivation [16], [17]. However, we couldn't supplement estrogen to relieve AIs-related musculoskeletal symptom, so finding good predictors is very important.

The effects of estrogen are mediated through binding to specific estrogen receptors (ER), which belong to the nuclear hormone receptor superfamily and are expressed in a number of cell types including osteoblasts, osteoclasts and bone marrow stromal cells [18], [19]. Two functional estrogen receptors have been identified so far, ESRa (ESR1) and ESRb (ESR2). ESR1 appears to be the major receptor, having a prominent effect on bone metabolism [20].

Progesterone and estrogen have synergistic effects on bone health, some clinical trials have found greater increases in spinal bone mineral density (BMD) when the progestin medroxyprogesterone acetate (MPA) is added to estrogens than with estrogens alone [21]. Progesterone must bind to the progesterone receptor (PGR) in order to exert its physiological effect.

To date, few researches involve SNP and AIs-associated MS-AEs. Mao et al [22] reported a TTTA repeat in CYP19A1 that was associated with AI-musculoskeletal symptoms. Ingle et al [23]identified that SNP of TCL1A gene maybe associated with MS-AEs in women treated with AIs. Since ESR1, ESR2 and PGR impact the fuction of estrogen and progesterone, we hypothesized that the presence of functional polymorphisms in these genes would be associated with MS-AEs among postmenopausal breast cancer survivors on AI therapy. To test this hypothesis, we performed a study of postmenopausal Chinese Han early breast cancer patients taking AIs to evaluate whether these polymorphisms were associated with patient-reported occurrence of MS-AEs.

## Materials and Methods

### Ethics Statement

Before the research was conducted, ethical board approval from the Third Affiliated Hospital of Harbin Medical University was obtained, and all of the volunteers provided written informed consent.

### Source of patients

From August 2007 to November 2011, we recruited postmenopausal Chinese Han women with completely resected stages I to III breast cancer in the third hospital of Harbin Medical University in Harbin that was estrogen receptor (ER) positive and/or progesterone receptor (PR) positive. Patients were currently taking letrozole 2.5 mg/day or anastrozole 1 mg/day adjuvant therapy for at least 6 months. Total 436 participants were enrolled and completed baseline questionnaires. The institutional review board of Harbin Medical University provided ethics approval.

### Case Definition for musculoskeletal adverse events

Cases had at least one of the following 6 MS-AEs since they had started AIs therapy: joint pain, muscle pain, bone pain, arthritis, diminished joint function, or other musculoskeletal problems. Cases were required to have at least grade 2 toxicity, according to the National Cancer Institute's (NCI's) Common Terminology Criteria for Adverse Events v3. Excluded from the study were patients who had inflammatory, metabolic, or neuropathic arthropathies or surgery of an afflicted extremity during the preceding 6 months were currently taking steroids (oral or injected) or narcotics.

Controls were completely free of any MS-AEs and off steroids (oral or injected) or narcotics for at least 6 months.

We selected scales that captured joint pain, stiffness, and functional status in the knees (Western Ontario and McMaster Universities Osteoarthritis Index [WOMAC]) and hands (Modified Score for the Assessment and Quantification of Chronic Rheumatoid Affections of the Hands [M-SACRAH]), a general pain scale used in cancer patients (BPI-SF) and a Functional Assessment of Cancer Therapy-General (FACT-G) [24].

### Specimen collection and DNA extraction

At the end of the interview, whole blood was taken for DNA isolation and genotyping. All samples were examined blind by laboratory personnel. DNA was extracted from peripheral blood samples of patients and controls using the AxyPrep Blood Genomic DNA Miniprep Kit (Axygen Biotechnology, USA). Each DNA sample was stored at −20°C until analysis.

### SNP selection

The known SNPs in the ESR1,ESR2 and PGR genes were selected using“Tagger” (http://www.broadinstitute.org/mpg/tagger/server.html) and data from the International HapMap Project (http://snp.cshl.org/). SNPs that “tagged” major Han Chinese in Beijing haplotypes and that were compatible with Illumina technology were genotyped. Since we were interested in major genetic effects rather than rare alleles, the goal of “tagging” was to find a set of tagSNPs in linkage disequilibrium with all SNPs in the HapMap data with a minor allele frequency ≥5%; the tagger program was ran in the aggressive tagging mode using 2-and 3-marker haplotypes with r^2^ and LOD thresholds set at 0.8 and 3.0 respectively. As a result, a total of 113 tag SNPs of ESR1, 13 tag SNPs of ESR2 and 7 tag SNPs of PGR, were identified.

However, this resulted in the identification of too many SNPs of ESR1 for our purpose. The purpose of the genotyped SNPs is to briefly reflect the genotypic polymorphism of ESR1 in our population, which is not the same as that of SNPs in genome-wide association studies or fine-mapping studies. Thus, we consider the SNPs in dbSNP http://www.ncbi.nlm.gov/SNP/ and available literature. The selection criteria were: 1) >10 % frequencies in the Chinese (CHB) population; 2)position in the gene or in the functional domains of the ESR1 protein and possible functional relevance; 3) the results from the previous genetic studies. The SNP rs9340799 located in intron 1 of ESR1 were also selected because it's associated with breast cancer in many studies.

### Genotyping

Genotyping of 27 SNPs was selected, the genotyping was performed by matrix-assisted laser desorption/ionization time-of-flight mass spectroscopy using a 384-format high-throughput Sequenom MassARRAY platform (MassARRAY Compact Analyzer, Sequenom, San Diego, CA, USA). Sequenom's SpectroDesigner software was used for SNP assay design, and SpectroTyper 4.0 was used to call genotypes automatically, followed by manual review.

We used PCR-based TaqMan assays (Applied Biosystems, Foster City, CA) as the secondary platform to check the results of genotype SNPs rs2234693 and rs9340799.

All assays were performed according to the manufacturer's instructions, and the results were analyzed on the ABI Prism 7500 using Sequence Detection Software (Applied Biosystems Co. Ltd., USA). In order to confirm the accuracy of the genotyping results, 10% samples of each SNP were randomly selected to be tested twice by different persons, the reproducibility was 100%.

### Statistical analysis

Differences between women who reported AIs-related MS-AEs and those who did not with respect to demographic, clinical, and treatment factors were examined with Χ^2^ tests. Comparing the scores of BPI-SF,M-SACRAH,WOMAC and FACT-G,t-test were used. Multivariate logistic regression models were created using presence or absence of AIs-related MS-AEs as the dependent variables, and demographic, clinical, and treatment characteristics as independent variables.

In 27 SNPs of ESR1, ESR2 and PGR by Sequenom MassARRAY assays, p values <0.05 were defined as nominally significant, and samples were further subjected to Bonferroni correction to correct multiple comparisons. We used Haploview 4.1 to construct haplotypes, linkage disequilibrium and estimate haplotype frequencies for both cases and controls [25]. Data were analyzed using SPSS 19.0.

## Results

### Predictors of AIs-Related musculoskeletal adverse events

The baseline questionnaires of 436 participants was showed in Table 1, presence of AIs-related MS-AEs was not associated with age, type of third generation AIs, duration of AIs therapy, ER/PR status, TNM stage, entry into menopause (natural v surgical/chemical), years since menopause or prior radiation therapy condition. Patients who had prior tamoxifen therapy were less likely to develop MS-AEs (p = 0.040; OR  = 0.607; 95% CI, 0.379–0.971) compared with those who did not. Importantly, patients who received prior taxane-based chemotherapy were more than two times than those who did not experience MS-AEs (p = 1.63E-4; OR  = 2.115; 95% CI, 1.429–3.130). Patients who had a low BMI (<25 kg/m^2^) and were obese (BMI>30 kg/m^2^) had nearly the same chances to experience AIs-related MS-AEs (OR  = 1.014, 95% CI, 0.388–2.651). However, women who were overweight (BMI, 25 to 30 kg/m^2^) were less likely to have AIs-related MS-AEs (OR  = 0.614, 95% CI, 0.406–0.926) compared with those who had a normal BMI.

**Table 1:** Clinical Characteristics and Treatment of Postmenopausal Breast Cancer Patients Receiving AI Therapy, by Presence or absence of AI-Related musculoskeletal adverse eventsevents group and control by PCR-based TaqMan assays.

Characteristic	Cases		Controls		p value	Odds ratio(OR)	95%CI
	(n = 206)		(n = 230)				
	NO	%	NO	%			
Age, years							
<55	42	20.4	52	22.6	0.640	1.0	
55–65	130	63.1	135	58.7		1.192	0.715–1.987
>65	34	16.5	43	18.7		0.979	0.454–2.111
Treatment arm							
Anastrozole	67	32.5	90	39.1	0.150	1.0	
Letrozole	139	67.5	140	60.9		1.334	0.900–1.977
Duration of aromatase inhibitor therapy, years							
<1	58	28.2	60	26.1	0.829	1.0	
1–3	120	58.3	135	58.7		0.919	0.577–1.467
>3	28	13.6	35	15.2		0.828	0.384–1.784
ER/PR status							
ER+/PR+	133	64.5	145	63	0.860	1.0	
ER+/PR−	58	28.1	65	28.2		0.973	0.625–1.513
ER−/PR+	15	7.3	20	8.7		0.817	0.373–1.791
TNM stage							
I	62	30.0	71	30.8	0.980	1.0	
II	128	62.1	141	61.3		1.04	0.675–1.601
III	16	7.8	18	7.8		1.017	0.402–2.582
Menopause							
Natural	12	68.9	152	66.1	0.530	1.0	
Surgical/chemical	64	31.1	78	33.9		0.878	
Years since menopause							
<10	82	39.8	91	39.6	0.929	1.0	
10–20	109	52.9	120	52.2		1.008	0.669–1.519
>20	15	7.3	19	8.3		0.876	0.363–2.117
Prior chemotherapy							
No	63	30.6	75	32.6	0.680	1.0	
Yes	143	69.4	155	67.4		1.098	0.733–1.646
Doxorubicin	80	38.8	93	40.4	0.480	0.932	0.768–1.132
Taxane	99	48.1	70	30.4	1.63E-04	2.115	1.429–3.3130
Prior tamoxifen							
No	171	83.0	172	74.8	0.040	1.0	
Yes	35	17.0	58	25.2		0.987	0.656–1.483
Body mass index(BMI), kg/m^2^							
<25	128	61.1	119	51.7	0.049	1.0	
25–30	66	32.0	100	43.5		0.614	0.406–0,926
>30	12	5.8	11	4.8		1.014	0.388–2.651

Note: AI  =  aromatase inhibitor; OR  =  odds ratio; ER  =  estrogen receptor; PR  =  progesterone receptor.

### Taxane based chemotherapy and AIs-related musculoskeletal adverse events

In AIs-related MS-AEs Group, 69.2% patients received a taxane-based chemotherapy.The results of Pain, Stiffness and Quality of life by taxane or non taxane-based chemotherapy are listed in Table 2. The mean pain score of BPI-SF was 5.5 and 3.0 for the taxane-based chemotherapy and non taxane-based chemotherapy groups, respectively, corresponding to a 1.83 deterioration in scores. Similar differences were seen for pain severity (5.9 vs 4.4; p = 4.93E-7), but not for pain-related interference (4.7 vs 4.2;p = 0.076).Similar findings were seen for the WOMAC and M-SACRAH subscales. Emotional well-being measured by the FACT-G showed a significant improvement for non taxane-based chemotherapy group compared with taxane-based chemotherapy group (18.1 vs 16.1; p = 0.004) (Table 2). However, no significant differences were observed for the FACT-G physical, social/family, and functional well being subscales.

**Table 2:** Pain, Stiffness and Quality of Life in AI-Related musculoskeletal adverse events Group by Taxane or non Taxane based chemotherapy.

Chemotherapy	Taxane	Non Taxane	p value	Adjusted* p value
	(n = 99)	(n = 44)		
	Mean(SD)	Mean(SD)		
BPI				
Worst pain(0–10)	5.5(2.2)	3.0(1.5)	7.00E-13	3.77E-11
Pain severity (0–10)	5.9(1.5)	4.4(1.6)	4.93E-07	
Pain-related interference (0–10)	4.7(1.4)	4.2(1.6)	0.076	
M-SACRAH				
Pain (0–200)	103(63)	54(26)	8.56E-10	2.15E-03
Stiffness (0–200)	112(73)	72(45)	1.01E-04	
Function (0–800)	293(135)	255(112)	0.082	
WOMAC				
Pain (0–500)	219(107)	189(89)	0.083	6.85E-07
Stiffness (0–200)	98(37)	42(20)	1.63E-22	
Function (0–1700)	605(285)	309(209)	1.24E-10	
FACT-G				
Physical well-being (0–28)	15.5(3.7)	14.4(3.2)	0.073	0.091
Social/family well-being (0–24)	17.6(5.2)	16.8(4.0)	0.318	
Emotional well-being (0–28)	18.1(3.8)	16.1(3.8)	0.004	
Functional well-being (0–28)	17.5(4.2)	16.5(3.6)	0.148	

Note: SD  =  standard deviation; BPI  =  Brief Pain Inventory; WOMAC  =  Western Ontario and McMaster Universities Osteoarthritis Index; M-SACRAH  =  Modified Score for the Assessment of Chronic Rheumatoid Affections of the Hands; FACT-G  =  Functional Assessment of Cancer Therapy–General.

* Adjusted for age, type of third generation AIs, BMI and prior tamoxifen therapy condition.

### SNPs and AIs-related musculoskeletal adverse events

The LD patterns of these three genes are shown in Figure S1. For ESR1 and ESR2 gene, there are no high LD were found in our selected SNPs; for PGR gene, only one block with high LD is identified with the size of 18 kb. The detailed information and genotypic frequencies of all the ESR1, ESR2 and PGR polymorphisms are shown in Table 3. The minor allele frequencies of rs2234693 and rs9340799 had statistical significant in genotypic distribution between the cases and the controls. The other 25 SNPs of ESR1, ESR2 and PGR showed no significant differences (P>0.05).

**Table 3:** Characteristics of Selected SNPs of ESR1, ESR2 and PGR.

dbSNP	Gene	Chr	Position	Function class	Minor Allele Frequency		OR	95%CI	p value	Bonferroni p
					Cases	Controls				
rs2234693	ESR1	6	152,205,028	intron	0.378	0.546	0.506	0.386–0.663	6.79E-07	1.83E-05
rs9340799	ESR1	6	152,205,074	intron	0.177	0.276	0.565	0.408–0.782	0.001	0.027
rs3020314*	ESR1	6	152,312,365	intron	0.197	0.200	0.981	0.703–1.372	0.913	
rs3798577	ESR1	6	152,462,823	UTR-3	0.398	0.415	0.931	0.710–1.221	0.607	
rs1801132	ESR1	6	152,307,215	cds-synon	0.461	0.507	0.834	0.639–1.088	0.181	
rs2228480†	ESR1	6	152,461,788	cds-synon	0.197	0.175	1.156	0.821–1.629	0.408	
rs2077647	ESR1	6	152,170,770	cds-synon	0.427	0.428	0.996	0.761–1.303	0.974	
rs1152580	ESR2	14	63764747	intron	0.485	0.474	1.047	0.803–1.367	0.734	
rs944459	ESR2	14	63769111	nearGene-3	0.311	0.428	0.922	0.693–1.227	0.578	
rs4986938†	ESR2	14	63769569	UTR-3	0.109	0.129	0.829	0.549–1.253	0.374	
rs1256064	ESR2	14	63770492	intron	0.481	0.474	1.027	0.787–1.340	0.844	
rs1256061	ESR2	14	63773346	intron	0.456	0.476	0.924	0.707–1.206	0.559	
rs8017441	ESR2	14	63785547	intron	0.097	0.111	0.862	0.557–1.350	0.506	
rs12435857*	ESR2	14	63793278	intron	0.313	0.314	0.998	0.749–1.330	0.988	
rs10148269	ESR2	14	63806677	intron	0.308	0.309	0.998	0.748–1.331	0.989	
rs1256031‡	ESR2	14	63815932	intron	0.498	0.522	0.908	0.696–1.185	0.476	
rs17179740	ESR2	14	63826504	intron	0.221	0.222	0.995	0.722–1.371	0.975	
rs1952586	ESR2	14	63829172	intron	0.192	0.193	0.989	0.706–1.385	0.948	
rs3020445‡	ESR2	14	63858397	intron	0.234	0.224	1.060	0.772–1.455	0.720	
rs1256120	ESR2	14	63874754	UTR-5	0.468	0.472	0.987	0.756–1.288	0.923	
rs11224556	PGR	11	100404823		0.318	0.317	1.003	0.754–1.334	0.986	
rs3740751	PGR	11	100406809	UTR-3	0.493	0.537	0.838	0.642–1.093	0.192	
rs561610	PGR	11	100408204	UTR-3	0.049	0.065	0.731	0.409–1.309	0.290	
rs471767	PGR	11	100410507	UTR-3	0.066	0.067	0.971	0.569–1.655	0.912	
rs572943	PGR	11	100460828	intron	0.257	0.265	0.960	0.709–1.299	0.790	
rs555653	PGR	11	100474755	intron	0.240	0.235	1.031	0.754–1.409	0.849	
rs537681	PGR	11	100493244	intron	0.163	0.172	0.937	0.656–1.338	0.719	

* controls  = 228, missing  = 2.

† controls  = 229, missing  = 1.

‡ cases  = 205, missing  = 1.

Further, we tested rs2234693 and rs9340799 by PCR-based TaqMan assays (Table 4).The results was similar to MassARRAY assays. rs2234693 and rs9340799 of ESR1 were associated with AIs-Related MS-AEs.TT genotype and the T allele in rs2234693 was statistically significantly lower in AIs-Related MS-AEs group than in controls (P = 0.001, OR  = 0.282, 95% CI, 0.147–0.540; P = 9.49E-7, OR  = 0.510, 95% CI, 0.389–0.669), however, CC genotype was higher (P = 7.79E-6). The frequency of AA genotype and the A allele in rs9340799 was higher (P = 2.20E-5; P = 3.09E-4), whereas AG genotype was lower (P = 2.24E-5, OR  = 0.446, 95% CI, 0.304–0.655) in AIs-related MS-AEs cases.

**Table 4:** Genotypes and Allele frequencies of 2 SNPs which had potentially statistical significance between AI-related musculoskeletal adverse events group and control by PCR-based TaqMan assays.

Reference SNP ID	Genotype	Frequency no.%)		p value	OR	95%CI	
		Cases	Controls				
		(n = 206)	(n = 230)				
rs2234693	CC	69 (33.5)	35 (15.2)	7.79E-06	1.0		
	CT	87 (42.2)	105 (45.7)	0.473	0.420	0.237–0.746	
	TT	50 (24.3)	90 (39.1)	0.001	0.282	0.147–0.540	
C		225 (54.6)	175 (38.0)	9.49E-07	1.0		
T		187 (45.4)	285 (62.0)		0.510	0.389–0.669	
rs9340799	AA	145 (70.4)	116 (50.4)	2.20E-05	1.0		
	AG	49 (23.8)	99 (43.0)	2.24E-05	0.446	0.304–0.655	
	GG	12 (5.8)	15 (6.5)	0.763	0.593	0.441–0.797	
A		339 (82.3)	331 (72.0)	3.09E-04	1.0		
G		73 (17.7)	129 (28.0)		0.553	0.399–0.764	

Further, haplotype CC of rs2234693 and AA of rs9340799 were genetic risk factors, therefore diplotypes which consisted of haplotype CC (rs2234693) and AA (rs9340799) were associated with AIs-Related MS-AEs risk (Table 5), although only diplotype CC/AG and CC/AA were statistically significant (P = 0.014;P = 5.94E-5). While, women with diplotypes (rs2234693/rs9340799) TT/AG had a significantly lower risk of AIs-related MS-AEs than did women with other diplotypes (P = 4.85E-7, OR  = 0.326, 95% CI, 0.203–0.525). Meanwhile in Table 6, high risk diplotype has more risks of AIs-Related MS-AEs (rs2234693/rs9340799: TT/GG, TT/AG, CT/GG and CT/AG) than low risk diplotype (rs2234693/rs9340799: TT/AA, CT/AA, CC/GG, CC/AG and CC/AA, P = 9.25E-8, OR  = 3.151, 95% CI, 2.050–4.843).

**Table 5:** Risk of AI-related musculoskeletal adverse events Associated with the Combination of Haplotypes at rs2234693 and rs9340799.

Diplotype	Cases (%)	Controls (%)	Genetic Risk Score	OR (95%CI)	p value
TT/GG	0 (0)	0 (0)	0		
TT/AG	19 (9.2)	65 (28.3)	1	0.326(0.203–0.525)	4.85E-07
TT/AA	31 (15.0)	25 (10.9)	2	1.384(0.846–2.265)	0.193
CT/GG	5 (2.4)	6 (2.6)	1	0.930(0.288–3.003)	0.904
CT/AG	17 (8.3)	30 (15.2)	2	0.633(0.360–1.113)	0.107
CT/AA	65 (30.1)	69 (30.0)	3	1.052(0.793–1.394)	0.726
CC/GG	7 (3.4)	9 (3.9)	2	0.868(0.329–2.290)	0.775
CC/AG	13 (6.3)	4 (1.7)	3	3.629(1.202–10.95)	0.014
CC/AA	49 (23.8)	22 (9.6)	4	2.487(1.560–3.965)	5.94E-05

**Table 6:** Risk of AI-related musculoskeletal adverse events Associated with Low-Versus High-Risk Diplotypes.

Diplotype	Cases (%)	Controls (%)	OR (95%CI)	p value
(rs2234693/ rs9340799)	n = 206	n = 230		
Low risk diplotype				
(TT/GG, TT/AG, CT/GG and CT/AG)	41 (19.9)	101 (43.9)	1.0	9.25E-08
High risk diplotype*				
(TT/AA, CT/AA, CC/GG, CC/AG and CC/AA)	165 (80.1)	129 (56.1)	3.151(2.050–4.843)	

* rs2234693 haplotype was CC or rs9340799 haplotype was AA.

### Discussion

AIs therapy is the standard of care for postmenopausal women with both early- and late-stage hormone-sensitive breast tumors [1]–[5].We found that among postmenopausal breast cancer patients taking adjuvant AIs therapy, about 50% of them reported musculoskeletal symptoms that either developed or worsened after initiating AIs. Previous researches were unable to assess mechanism-specific predictors of development of toxicity. Therefore, we focused on nonmechanism based, clinical and genetic predictive factors of toxicity, which could be used to guide individualized treatment decision making at the time of endocrine therapy initiation or to direct specific modifying interventions as they become available.

Our observation that low BMI or obese, prior nor tamoxifen therapy and prior taxane-based chemotherapy patients who were more likely to report AIs-related joint symptoms. Among these predictive factors, we found patients who received taxane chemotherapy were more than two times more likely than other patients to have AIs-related MS-AEs.The finding was consistent with prior reports [6], [26]. Chemotherapy neurotoxicity is a side effect of several agents used in the chemotherapy treatment. Sometimes, it's difficult to distinguish with AIs-related MS-AEs.Hence, we further analyzed the pain, stiffness and quality of life through questionnaires. We found the patients who had Taxane-based chemotherapy not only had more risk of AIs-related MS-AEs, but also experienced a more serious adverse symptom.The results were confirmed by BPI-SF, WOMAC, and M-SACRAH questionnaires.

Excecpt for clinical characteristics, recently, a genome-wide nested case-control study had identified four SNPs in tight LD on chromosome 14 that were associated with MS-AEs in women receiving AIs for resected early-stage breast cancer [23].In this study, two ESR1 SNPs, rs2234693 and rs9340799 in the first intron were associated with AIs-related MS-AEs. Of note, rs9340799 and rs2234693 are in linkage disequilibrium in Caucasian and Japanese [27]. A meta-analysis of studies published through 2001 concluded that available data did support a relation between ESR1 rs2234693 and BMD [28]. We observed that ESR1 rs2234693 CC genotype was associated with higher risk of AIs-related MS-AEs.The results were similar to previous studies. For Chinese women, the CC genotype of rs2234693 was conferred a high risk of osteoporotic vertebral fracture, hip fracture [29], and decreased levels of BMD for hip, spine or whole-body regions [30]; for premenopausal Caucasian and Italy women, ESR1 rs2234693 C allele presented lower LS-BMD and TH-BMD than TT genotypes [31].

rs9340799 was another SNP of ESR1 commonly studied in relation to bone outcomes, although most of them was not statistically [31], [32]. For postmenopausal women, the effect of LTM on femoral neck BMD was significantly larger for those with the AG/GG genotype of ESR1 rs9340799 than for those with the AA genotype [33]. In our study, AA genotype of rs9340799 was the risk factor of AIs-related MS-AEs, yet AG genotype was the protective factor. Therefore, we got the results that genetic polymorphisms in ESR1 play a major role in the etiology of AIs-Related MS-AEs. The results of diplotypes analysis showed that patients who had haplotype CC (rs2234693) or AA (rs9340799) were more than three times to experience AIs-related MS-AEs than those who did not.

In fact, functional genomics studies of polymorphisms remain a weakness. Except for introns [34], [35], few polymorphisms in the other region of ESR1 were associated with bone health. The SNP rs2941740 (allele T) was associated with hip BMD among East-Asians population [36]. Japanese women with the ESR1 rs3798577 CC or TC genotypes had lower LS-BMD than did Japanese women with the TT genotype [32]. Another study in China indicated that rs6929137 was an osteoporosis susceptibility SNP [37].

To our knowledge, few research had been examined the relations between bone health and SNPs in the ESR2, while no possible relations in PGR gene previously.SNP rs1256120 in ESR2 was reported to be associated with Adolescent idiopathic scoliosis predisposition and curve severity [38]. Another research detected a significant association of rs960070 in ESR2 with hip fractures in 700 elderly Chinese subjects [33]. Unexpectedly, no evidence of association between AIs-related MS-AEs and SNPs in ESR2 and PGR was found in this study.

### Limitations

One limitation of this study is the limited number of samples, a great number of postmenopausal early breast cancer patients in Harbin of China choose tamoxifen, not aromatase inhibitors as auxiliary endocrine therapy because of economic reasons. In addition, we did not obtain biological samples such as synovia or muscular tissue in order to find other useful predictors, because it was always refused by breast cancer patients and their families. Such data might help to find the clinical and genetic predictors to the aromatase inhibitors related musculoskeletal adverse events.

### Conclusions

In summary, AIs-related musculoskeletal adverse symptoms might be exacerbated by prior taxane therapy. The determination of the mechanism of MS-AEs would enable a focused approach to amelioration of symptoms, thus facilitating compliance and improving the benefits of AIs for women with early breast cancer.The genotyping result identified two SNPs rs2234693 and rs9340799 in ESR1 that were related to MS-AEs in patients receiving AIs adjuvant therapy. Future functional studies would explore possible biological mechanisms underlying the associations of genetic risk or protective infactors of AIs-related MS-AEs.

## Supporting Information

Figure S1 Linkage disequilibrium between genotyped SNPs of ESR1(A), ESR2(B) and PGR(C).
(TIF)
