# Supplementary figures and images for: Indications of Clinical and Genetic Predictors for Aromatase Inhibitors Related Musculoskeletal Adverse Events in Chinese Han Women with Breast Cancer
Source: PLoS One. 2013 Jul 19;8(7):e68798. doi: 10.1371/journal.pone.0068798 (PMC3716812; doi:10.1371/journal.pone.0068798)

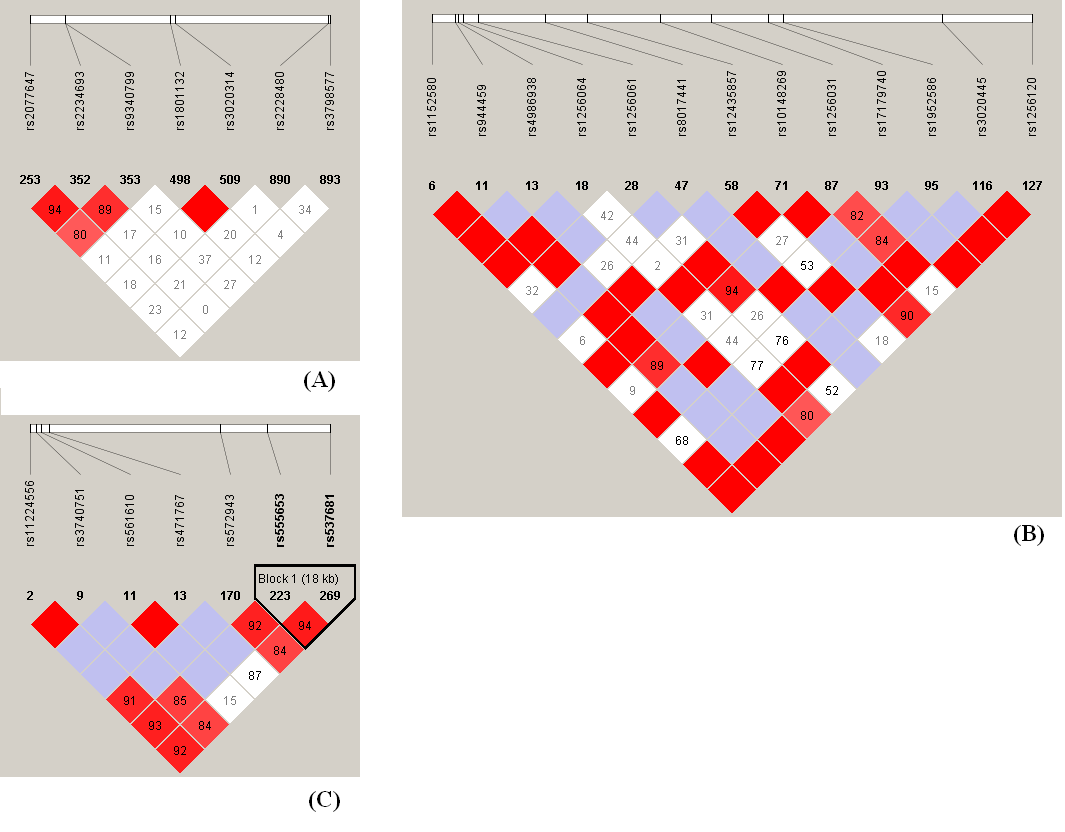

Supplement: Figure S1 — Linkage disequilibrium between genotyped SNPs of ESR1(A), ESR2(B) and PGR(C). (TIF) [file pone.0068798.s001.tif]
